# Abstracts and Gleanings

**Published:** 1881-05-20

**Authors:** 


**Treatment in Cases of Excessive Lochlal Discharges.**—Dr. Hugh Miller, in a clinical lecture delivered at Glasgow, recommends the following prescription in cases in which there is an excessive discharge, accompanied by a relaxed condition of the uterus. He administers one drachm doses of liquid extract of ergot repeated every three or four hours, and

℞ Quinia sulph.............................................. ½ drachm.

Acidi hydrobrom.......................................... 6 drachms.

Aquæ ad.................................................... 2 ounces.

Dose, one drachm in aq. ter. in die.

By this method large doses of quinia may be given without causing headache. In septic cases Dr. Miller advises the employment of sulpho-carbonate of potash, in the form of powders, in doses of ten to fifteen grains internally three times a day.

When the discharge is suspended, the treatment consists of turpentine stupes applied over the lower part of the abdomen, with the addition of warm moist cloths, or of sponges, pressed out of hot water, and applied to the external parts. In special cases, which require an antiseptic form of treatment, Dr. Miller makes use of a solution of thymol, one part to five hundred parts of water, or, better, three grains of thymol to an ounce of eau de Cologne. This mixture, which has a pleasant and rather refreshing odor, is simply sprinkled over the napkins before they are used. In severe cases, with a putrid odor, a solution of permanganate of potash, injected with Higginson's syringe, provided with a vaginal portion, is made use of; the injection of the fluid is continued till it returns unaltered in color. In all cases where the discharge is excessive, tincture of arnica is employed; the tincture is used in the proportion of one teaspoonful to a cupful of water; it acts as a mild astringent and disinfectant.—*Canada Medical Record.*

### ABSTRACTS AND GLEANINGS.

### NEW REMEDIES.

The following extracts are from an article by Dr. H. G. Barrow, in Chicago Medical Journal:

**The Sugar of Milk.**—In Dr. Talmy's new work, published in Paris, he prescribes for the diarrhœa of hot countries, from 20 to 200 grammes of sugar of milk daily. He administers it in the simplest way; the sugar is dissolved in a little water, or as a draught in the course of the day. The dose of sugar to be taken is put into a small quantity of milk, according to the habits and the digestive capacities of the patient. The treatment is spread over several months, diminishing the dose as nutrition becomes more considerable and easy; according to the published work referred to, the endemic diarrhœa of hot climates is the result of a functional lesion of the liver, which results in the diminution and even the suppression of the glycogenic function of the liver. The sugar of milk may thus replace the glucose which is wanting in the blood.

**Pilocarpin.**—The Druggists' Circular says: A substance has been separated from the leaves of the *pilocarpus pinnatus*, or jaborandi, that seems to possess all their virtues; it has been called *pilocarpin*. It has a semi-fluid consistence, has a yellowish color, an agreeable odor, is free from acidity, and is spoken of as an alkaloid. In doses of one-twelfth to one-fourth of a grain, it is equal to an infusion containing the strength of from five to ten grains of the leaves. It has but little effect on the heart's action, or on the temperature of the blood, nor does it act as a narcotic on the brain. Jaborandi is, in truth, a harmless but highly potent medicine, and its efficacy lies in its action as a sialagogue and diaphoretic.

**Ether Spray.**—In a serial known as "The Ameriecan Practitioner," Dr. W. H. Griffiths reports two cases of severe post-partum hemorrhage, in which every known means had been adoped unavailingly, and to which he was called in consultation. He says, it flashed across my mind in the first case to try the effect of ether-spray, and accordingly I directed a large spray over the abdominal walls along the spine, and over the genitals; the uterus at once responded, and the cessation of the hemorrhage was almost immediate. In the second case, I lost no time in adopting a similar treatment, and with an equally successful result. The doctor further says, I have consulted several eminent obstetric practitioners in Dublin, and am informed by them that they are not aware that this treatment has been heretofore proposed. The advantages of ether-spray over the application of cold water, and the other means usually adopted in these cases must be apparent to every practitioner of midwifery.

**A New Use of an Emetic.**—A physician makes known to the profession, through a medical publication, a simple means of arresting obstinate epistaxis, rebellious to all other treatment. His case, he says, resisted all the means usually resorted to for arresting such hemorrhages, mustard foot baths, cold ice to the nucha, plugging of the nasal orifices, elevation of the arms, injection of the perchloride of iron as practiced by Crequy, besides other means. If the patient be not already enfeebled, fainting spells will soon come on if the hemorrhage continues. What is to be done? A simple means has succeeded in my hands, a light emetic quickly administered, soon provokes nausea, then vomiting, and the hemorrhage is incontinently arrested.

**Sulphur and Sugar.**—In the convulsive cough that often precedes measles, M. Tourtual recommends sulphur and white sugar, mixed in equal parts; of which half-teaspoonful doses are to be given occasionally. This is a very safe and simple remedy, and from the high source whence emanates the prescription, and from the assurance that it is effectual in alleviating the cough, certainly it is worthy of a trial.

**Damiana.**—This is a new agent that has recently made its appearance; it has not existed long enough to receive a botanical appellation. Damiana is the name by which this plant is known on the western coast of Mexico, where it grows. As yet, I believe, but little is known of its medicinal properties, as its use has been somewhat limited.

Dr. J. J. Caldwell, of Baltimore, has given this new remedy a number of trials, and he thus speaks of it: "I am well satisfied, from quite an extended experience with the tincture and extract of this plant, of its powerful influence over the urino-genital organs of both sexes, as in moderate doses it increases the flow of urine, as well as the sexual appetite. The doctor reports several cases where its powerful aphrodisiac effects were obtained after the usual remedies, such as strychnia, phosphorus and electricity, had failed.

**Neuralgia.**—The attention of the medical profession in Paris has been called to the treatment of "neuralgia" through the nasal passages. Mons. Raimbert made a communication to the Academy of Sciences upon the use of a mixture of powdered white sugar twenty grains, to morphine one grain, used as a snuff, which is affirmed to be effectual in the treatment of dental, sub-orbital and frontal neuralgia. It is also applicable to all kinds of headaches.

**Arsenic in Consumption.**—M. Montigny, French Consul in China, in reference to the use of arsenic by the Northern Chinese, says, they mingle it with their smoking tobacco. According to missionaries who have lived a long time there, tobacco free from arsenic is not sold. The same witnesses assured the Consul that the arsenic smokers were stout fellows, with lungs like blacksmith's bellows, and as rosy as cherubs. The publication of Montigny's statement called out a letter from Dr. Fonde, who announced that some years ago in the course of a discussion at the Academy of Medicine, on the agents to be employed to cure tubercular consumption, he told the assembled doctors that he found but one successful means of combatting this dreadful disease; that means was the smoking of arsenic. The doctor re-affirms his commendation of this remedy.

**The Sulphur Ferri in Suppuration.**—The Boston *Journal of Commerce* reports this remarkable case. A child burned all over was recently brought into the hospital, the suppuration from the wounds so profuse, that the ward in which he lay was almost uninhabitable. He was placed in a bath containing two handfuls of sulphate of iron, the cessation of pain was almost immediate; after repeating the bath twice a day, for fifteen or twenty minutes at a time, the suppuration moderated, the fœted odor disappeared, and the patient rapidly recovered.

**Carbolate of Ammonia.**—Dr. Declat has lately urged with much earnestness the virtues of the carbolate of ammonia in the treatment of malignant pustule. This substance is applied first as a caustic, and then administered internally in a dose from fifteen to thirty grains in twenty-four hours. In one instance four butchers were attacked with malignant pustule, derived from infected cattle, and two were attended at home, while the other two were taken to the hospital, and placed under Dr. Declat's care, and were treated with the carbolate of ammonia, as above described. These were entirely cured in a reasonably short space of time, while the others, who were treated at home by the ordinary method, succumbed to the malady.

**Puerperal Mania.**—The following case was reported in the *Virginia Monthly:* Dr. Liebman attended a lady seized, on the ninth day after her confinement, with mania. The delusion assumed the character of extreme hatred of her very near friends. He ordered chloral hydrate in ten grain doses, repeated every two hours. After the exhibition of a few doses, sleep was induced, and by the next morning she was restored to reason. Except a slight nervous trouble, she entirely recovered at the end of three weeks.

**Small Pox.**—An Englishman of some note sent to a Liverpool paper this remarkable statement, viz: The worst case of small pox can be cured in three days, simply by the use of cream of tartar. The formula is, dissolve an ounce of cream of tartar in a pint of water, which is to be drank cold, at intervals. The writer of this affirms that it is a certain remedy, that it has cured thousands, never leaves a mark, never causes blindness, and avoids tedious delaying. If this is a reliable statement, it is very remarkable, and certainly deserving of a trial.

**Hay Fever.**—Prof. Bins, of Bonn, writing on the subject of "hay fever," first calls attention to the discovery made by Helmholtz as far back as 1868, of the existence of uncommon low organisms in the nasal secretions in this complaint, and of the possibility of arresting their action by the local employment of quinine. Helmholtz having been made aware of the poisonous action of quinine upon infusoria, determined to make an experiment with that substance on the vibrionic bodies he had discovered in the nasal secretions of persons suffering from hay-fever, and for that purpose he employed a weak solution of quinine, which he injected into both nostrils. The result was most satisfactory, and the cure which took place in his own case, induced him to try the remedy upon two of his patients, with like satisfactory results.

Dr. Frickhofer and Prof. Busch have succeeded in curing this affection by the same method. Prof. Bins says, after due care has been taken to have quinine pure, that a tepid solution should be used, and a proper nasal syringe should be the medium through which to apply it.

---

### SNAKE-BITE.

EDITOR MEDICAL BRIEF :—Enclosed you will find a cure for snake-bite which I have seen my father use in Alabama years ago to cure snake-bite, also spider bites. The horehound is not the tame that grows about old fields, but is the wild, that has a single stem about four feet high; the leaves grow opposite each other alternately, and resemble the tame horehound; also, has a white blossom in clusters resembling the boneset (eupatorium perforliatum), and grows about pine slushes.

### ALMOST A CENTURY AND A-HALF.

*Editor Home and Farm:*—I have copied the following exactly, save the old style of f's for s. If you think it worthy a place in "Home and Farm," well and good. I can send you more from the same source.

Pactolus, Pitt Co., N. C. MRS. J. J. R.

*Introductory Letter—From the Carolina Gazette, May* 9, 1749.

*To the Printer:* Sir—I am commanded by the commons house of assembly to send you the inclosed, which you are to print in the *Carolina Gazette* as soon as possible. It is the negro Cæsar's cure for the bite of a rattle-snake; for discovering of which the General Assembly hath thought fit to purchase his freedom, and grant him an allowance of £100 per annum during life.

I am, etc., JAMES IRVING.

*Negro Cæsar's Cure for the Bite of a Rattle-Snake.*

Take the roots of plantain or horehound (in summer roots and branches together), a sufficient quantity; bruise them in a mortar, squeeze out the juice, of which give, as soon as possible, one large spoonful. If the poison is swelling, it must be forced down the throat. This will generally cure. If the patient finds no relief in an hour after, give another spoonful, which never fails. If the roots are dried, they must be moistened with a little water. To the wound may be applied a leaf of good tobacco, moistened with rum.

The above was written by my father, Dr. F. L. Meriwether, of Daly, Houston County, Texas, just before his death, which occurred February 9, 1881. He was an eye witness to the efficiency of the remedy, and had written the above for the purpose of having it published in your journal.

J. B. COX, M. D., *In Medical Brief.*

**Expert Testimony in Toledo.**—Much has been written on this subject during the past few years, and much discussion had, but until recently no one has been found who had the courage of his convictions to bring the matter to a test.

In a case recently tried in this city, State vs. Hakeos, indicted for the killing of one King by a pistol-shot, Drs. Biglow and Brigham attended the wounded man, and after his death made the post mortem examination for the Coroner. Being subpœnaed as witnesses for the State, they gave testimony to all things of which they had knowledge, but being interrogated as to what, in their opinion, was the cause of death, they asked to be excused on the ground that this was expert testimony, and that they had not been paid, nor were to be paid as expert witnesses. The following is the account of the proceedings as reported stenographically in the *Telegram* of Dec. 4. The witness is Dr. Brigham, but the facts are equally applicable to Dr. Bigelow, who was on the stand the previous day:

Mr. Ford—Doctor, from your observations at the office and at the house, and from your previous medical experience, what is your opinion of the cause of the death of this man King?

Witness—That is a question I do not see that I must answer.

Mr. Ford—What are your grounds for wishing to be excused?

Witness—Well, it is an opinion that demands a good deal of consideration.

Mr. Ford—You got paid by the county for making a post mortem examination, did you not?

Witness—I haven't got the pay yet. (Laughter.)

Mr. Kennedy—If we are entitled to an answer, we'd like to have it in the present case and at the present time.

The Court (Judge House)—We are now asking what was the cause of the death of this man. Now the truth is, all the facts have been given in the case that came under the witness's general attention. You now want to ask him of his opinion. Suppose he were to state his wound were not the cause of the death of the boy, would any one believe him? Now this matter is for the jury to determine. They have the facts before them. The question is one that may be asked, and it is a legal question. But it is calling upon these gentlemen for their opinion, which is the result of money and study and deep thought for many years, and asking them to do all this for a mere pittance of seventy-five cents a day. It is wrong, there is no question about it. Practically, they are right. The law has not provided for this thing, it is a *casus omissus;* it is a thing which, if the attention of the Legislature had been directed, would have been provided for, because it is just and right. There is no more right in calling upon these medical gentlemen for their years of labor and study and expense, than there would be to call upon a lawyer. Now suppose a man has an important matter on which he wants advice, and takes it to one of the lawyers here, and says: "I want your legal opinion;" is there any respectable lawyer who would consent to look after this matter without charging for giving his opinion? Well, now, it is precisely that sort of opinion that our medical men are called upon to give. They are entitled to fair and respectable compensation for that opinion, and they ought not to be compelled, in my view, to give the result of their observation and thought, and when it comes from all these years of labor. I therefore will not force that question unless witness sees fit voluntarily to answer it. In this instance the power of the law might be used, but it would be to do what is substantially wrong, asking from a man a thing that is valuable and that there is no compensation for. I will therefore leave the matter, so far as opinion is concerned, with them.

Mr. Kennedy—We have no objection to the doctor being paid for his evidence.

The Court—Neither the prosecution nor the court have any power over the public treasury.

Mr Kennedy—It is a question we have a right to demand an answer to, and a right to ask.

The Court—It is a question you have a right to ask; but I will not use the arm of the law, under the circumstances, to compel an answer to it.

Mr. Ford—I wish to say a word on this point.

Of course this is an important matter as far as the administration of public justice and law is concerned. Mr. Kennedy and myself are simply agents of the State in this case, and have to discharge a duty toward the public, and deeming this an essential ingredient to make out this case, we feel we cannot discharge our duty to the public without insisting on an answer to this question. And I would have it understood that if the case fails, and if justice is not done, the responsibility rests, not upon us, but upon the decision made by the court.

The Court—You are simply doing your duty, gentlemen, in asking the question, and it is a proper question; but I will not compel an answer by using the arm of the law in the case until such action as the Legislature may see fit to take in the matter.

It will thus appear that the witnesses owed their immunity from committal for contempt to the leniency of the court, who, in our judgment, took the only rational and just view of the question. It is true that, in all probability, the opinions of these witnesses as to the cause of the death could have had no weight with the jury. It does not require a professional or scientific dictum to make it apparent that a pistol bullet which perforates a man's abdomen from back to front and spills the contents of the intestines into the peritoneum, is the cause of that man's death. The fact that his assailant was promptly found guilty by the jury, is conclusive on that point. How far the judge's desire for fair dealing might have carried him if the case had really required such elucidation as only expert testimony can furnish, remains an open question. At all events, the principle, as expounded by the honorable court, is a just one, and we wish to express our indebtedness to Drs. Bigelow and Brigham for its education.—*New Orleans Medical and Surgical Journal.*

**Parke, Davis & Co.**, of Detroit, Michigan, in an article to the *College and Chemical Record*, make a liberal proposition relative to the testing of new drugs. They say that the unsupported testimony of the most careful and conscientious scientific investigation cannot be accepted as conclusive evidence; but the accumulated results of the extended experience of many competent observers is the only safe criterion to guide the physician in the treatment of the sick. If the profession had waited for an accumulation of this kind, however, before employing new drugs, the properties of rheubarb, cinchona and opium would never have been known. It should be the purpose, therefore, of trade, as well as science, to do all in her power to facilitate experimentation for the purpose of clearing up all representation regarding new drugs, and coining it, as far as possible, into a definite scientific literature! With this intent we have adopted the following plan, suggested by Dr. Stewart, and recognizing the benefit its adoption must be to trade by increasing the demand for new drugs, we offer our aid to the profession in carrying it out.

The plan suggested is to treat the patients in the numerous hospitals and dispensaries throughout the country with drugs which have proved themselves of value, and report the results to the medical press. The collection of these reports would furnish, in a short time, as much material as was procured by older methods in a century, and from them could soon be compiled a valuable literature. Though these reports benefit us only directly, and to the extent that we are identfied in the introduction of the drugs or its sale, we offer to the hospitals, gratuitously, drugs for this test, and we do not even request that our names shall be used in the journals in connection with the work.

And, finally, it is to be hoped that the medical profession will give us credit for the integrity of our motives in the introduction of new remedies from the platform on which we stand, and because of the methods which we have adopted.

**Ethylate of Sodium in the Treatment of Nævus.**—In the *London Lancet*, Dr. Richardson reports a number of cases of nævi removed with this agent. We extract two of the cases:—The first was an instance of the common form of nævus on the scalp in an infant three months old. After perfect recovery from vaccination, the treatment commenced in the usual way by the application of ethylate over the growth, by the means of the glass-rod. The nævus was small, not larger than a fair-sized hazel nut. The first application caused a dense scale to form, which was loose and removable on the fifth day. The ethylate was then re-applied, and five days later, when the new scale was removed, the nævus was reduced to the size of a small bean. It remained in this state during three further applications of the ethylate, being much longer under treatment than I had expected after the second application. On the seventh application it was nearly removed, and one additional touch a fortnight later completely removed it. No constitutional symptoms interfered with the course of the treatment, and no scar remained.

In the next example the treatment was almost identical, both in respect to mode and to result; but as the patient was very restive and screamed extremely when the ethylate was applied, advantage was taken to make the application when the infant was in deep sleep. The plan succeeded so well that I ventured to suggest its general adoption in young children whenever the nævus is in a situation where it can be easily got at, and whenever an intelligent nurse or parent can be taught to make use of the solution in a safe and efficient manner. In the case in question the nævus was quite removed in the course of six weeks, and it can scarcely be said that any pain at all was inflicted. No scar has been left.

**Typhoid Fever.**—Dr. Bristowe considers the treatment of enteric fever under four heads: 1. Diet; 2. Medicine; 3. Alcohol; 4. Baths; and in concluding his paper says: "Let me state briefly the treatment to which I should like to be subjected if ever, unfortunately, I should become affected with enteric fever. I should like to be placed in a cool, well-ventilated room, and covered lightly with bedclothes; to have a skillful and attentive nurse to look after me; to be fed solely with cold milk, unless vomiting should demand the addition to the milk of medicine calculated to allay vomiting. If diarrhœa became troublesome, or ever there was much pain or tenderness in the cœcal rings and in the bowels, I should like to be treated, not with laxatives, but with opium, given either by the mouth or the rectum. If constipation were present, I should, excepting in the first week, like to have enemata only for its relief. In the event of intestinal hæmorrhage coming on, I should like to have ice to suck or ice-cold fluids to drink, cold compresses to the belly, and cold injections into the bowels; and, though I am skeptical as to their efficacy, I should still choose to have astringents, and more especially lead, given to me at short intervals. If perforation should take place, let me have large and repeated doses of opium. Stimulants I should prefer to be without early in the disease; later, however, and during convalescence, I should like to have them in moderation. As to the cold baths, I would rather not have them; but I would, nevertheiess, leave it to my physician to exercise his discretion in the matter. I would leave it also for him to decide, according to circumstances, whether alcohol should be administered to me in large quantities. I should prefer not to be treated at a temperance hospital."—*Buffalo Medical Journal.*

**Coca in the Opium and Alcohol Habits.**—Dr. H. F. Stimmell, Chattanooga, Tenn., in *Therepuetic Gazette*, says:

Having put the fluid extract of coca (coca erythroxylon) to a very severe test, I am prepared to give you the result of my experience. To say that I am surprised or astonished at the wonderful, and almost incredible effects of that new remedy as a nervous stimulant would not adequately express my appreciation of it. I will report a case:

1. Mr. X. Y. had been addicted to the habit of taking morph. sulph. for about five years, commencing with one-eighth gr., for lumbago, changing it from internal to external application (hypodermically over lumbar region), and gradually increasing the quantity until he reached the enormous dose of twenty-five grs. as a maximum, three to four times a day. His nervous depression became so great that he could not hold his pen, or button his shirt, or handle knife and fork at breakfast, without taking his usual dose directly after rising. He suffered from all the consequences of the drug. His mind became deranged, and he even attempted the life of his wife and children, after which, believing he had succeeded, he swallowed one drachm of morphine, followed by a five-ounce dose ot paregoric with suicidal intent. I was called sometime after but found him suffering scarcely any from the effects of the drug, and the only treatment consisted in keeping him in motion. After his complete recovery I talked freely with him regarding his infirmity, and promised to cure him if he would pledge himself to buy all of his morphine from me, thus enabling me to control his doses. I started him with the allowance of three twenty-grain doses of morphine to be taken with a drachm of coca. In a week his morphine allowance had decreased to ten grs. a day and his dose of coca increased to one-half ounce, and now, three weeks after commencing this treatment, the morphine has been entirely suspended. Yesterday his wife came to my store handing me a package ot powders of morph. sulph., labeled and dated by me for her husband, in which I had confirmatory evidence of his assertion of abstinence.

**Chlorate of Potassium.**—Dr. Landesberg, in *Medical Bulletin*, draws attention to the use of chlorate of potassium in cancroid affections. As a topical remedy in carcinoma, it was first tried by Tedeschi, as early as 1847, with such marked effect that his procedure soon found enthusiastic supporters, especially among the French profession. The communication of Bergeron, Milon, and Blondeau in the *Union Medicale*, of 1865, No. 154, gave full credit to the efficacy of chlorate of potassium in the treatment of cancroid. Their method consisted in applying to the ulcerated surface either a four per cent. or a concentrated solution of the drug. In a case of scirrhus of the nose, in a woman of 84 years, Bergeron observed a perfect cure, after a treatment of four months. The cicatrization started from the edges of the ulcer; the latter healed entirely, leaving flat, white, solid cicatrix. A year later, Debout published in the *Bulletin de Therap*, lxvi., January 15, 1864, the remarkable results of his experience, with the use of chlorate of potassium, in cancroid affections, which were fully corroborated by the subsequent publications of Leblane, Cooke, Charcot, Delpech, and Michon. The local application of chlorate ot potassium was combined by Charcot with the internal use of the drug, in daily doses of 30 grains.

**Anthracæmia—Wool-Sorters' Disease.**—Dr. W. R. Blackwood, of Philadelphia, contributes to the Philadelphia Medical Times, an interesting paper on this disease, which has attracted especial attention in England. A committee appointed from the Bradford Medico-Chirurgical Society reported the result of four typical cases, with the results of *post-mortems*. They seem to leave no doubt of the fact that this affection is due to blood-poisoning by the so-called bacillus anthracis, a low form of bacteria, presumably contaminating the wool, and which gains entrance to the blood of those affected through the likeliest channels—the lungs or stomach. The symptoms so far recognized are violent cephalalgia—often unilateral—fever, intensifying in its progress, severe pleuritic pain, crepitant inspiratory rales, and finally profuse diarrhœa. The differential diagnosis between anthacæmia and typhoid fever is clear, and ordinary care only is requisite to distinguish them. Cases have been complicated with malignant pustules from inoculation by scratching pimples or abrasions, especially about the face, and in such instances the neighboring lymphatics have become greatly enlarged. The prognosis is bad, and treatment is as yet apparently unsettled. *Post-mortem* investigations show softening of the bronchial glands, and accumulations of fluid in the pleural and peritoneal cavities. The intestines, beyond injection and low inflammatory signs, give no evidence. The glands of Peyer are not softened or ulcerated. Bacillus is abundant in the fluids of the closed cavities, in the viscera and in the blood. Inoculation of blood containing this form of bacteria in the lower animal, as tested in the mouse, rabbit and guinea-pig, produces the disease, death supervening in from thirty-six to seventy-two hours. Decomposition is rapid, especially at the site of puncture in the case of inoculation. Dr. Blackwood reports two typical cases, occurring in his practice recently.—*Va. Med. Monthly.*

**Bi-Meconate of Morphia.**—Dr. T. B. Curtis, in Boston Medical Journal, called the attention of the Boston Society for Medical Improvement to this preparation of morphia, which he believed to possess advantages rendering it superior, for certain exceptional cases, to all other opiates. Morphia, in its natural condition in the poppy, is combined with meconic acid.

The bi-meconate was introduced by Squire, 1839, in the form of a solution of bi-meconate of morphia, which was said to be of the same strength as laudanum, and to possess in an eminent degree the sedative powers of morphia, but having this superiority, that it disturbed the head, bowels and stomach less than any other preparation of opium. For hypodermic use, the solution, according to Squire, could be evaporated to one-twentieth of its bulk, so that three minims were equal in power to a half grain of acetate of morphia. In prescribing this salt, it should be borne in mind that it contains only half as much morphia as an equal weight of the sulphate. To obtain similar effects to those of the sulphate, therefore, a double dose must be given.

Dr. Curtis had recently met with three patients, who were inconvenienced in various ways by opiates to such a degree that, although suffering much pain and distress, they were debarred from using these means of relief. One was an elderly lady, who had for some weeks been affected with protracted hepatic colic and jaundice, due to gall-stones, and followed by severe hepatic pain and nausea. Opium and morphia caused intolerable nausea and vomiting; but the bi-meconate was taken in fair doses, with little or no inconvenience, and was productive of much relief and benefit.

**Digitalis in Scarlet Fever.**—Regarding the treatment of this common disease, Dr. William B. Atkinson, of Philadelphia, writes in Medical and Surgical Reporter: In my own experience, no single remedy has given me such good and such constant results as digitalis. About the year 1858, Dr. Lewis P. Gebhard read a paper before one of our medical societies, very strongly advocating the use of this article in all forms of this disease, and claiming for it the character of a specific. His method was to put one drachm of the powdered leaves of digitalis to twelve tablespoofuls of boiling water, and, when the infusion had cooled, to give it in teaspoonful doses every hour, according to the age of the child and gravity of the symptoms. Since that time, I have used it in a large number of cases, and with the best results. I generally order it prepared in the same way, and direct the nurse to give it in teaspoonful doses every hour or two, until the pulse and temperature are positively reduced; and then to lenghten the interval so as to matntain the effect thus obtained. I believe that I have almost invariably observed the symptoms to moderate within from twelve to twenty-four hours, and I feel confident that while I have never in a single instance known any of the so-called poisonous effects of the remedy to follow, I have also failed to see the usual dangerous sequelæ in many cases, and only slightly in any.

**The Treatment of Itch.**—A writer from Paris says that at present itch is cured in one hour and a half, at the St. Louis Hospital. The first half-hour the patient, absolutely nude, rubs himself from head, or rather neck, to foot, with soft-soap. The second half-hour he is put into a tepid bath, where he continues the soft-soap frictions. The third half-hour he rubs his body with Helmerich's sulpho-alkaline ointment. He puts on his clothes without washing off the ointment, so as to keep it in contact with the surface for twenty-four hours. While the patient is treating himself, his clothes are purified in a specially constructed stove, at a temperature of 120°, and exposed to sulphur vapor. Four thousand itch patients are treated here annually.

The hospital treatment is a rough one, and sometimes causes attacks of eczema. It may be mitigated thus: toilet soap is substituted for soft-soap, and Hardy's modification of Helmerich's ointment used, lard one hundred parts,i sulphur sxteen parts, bicarbonate of potash eight parts, by weight. The patient should have his sheets and all under-linen changed immediately.—*Medical and Surgical Reporter.*

**Asthma.**—In attempting to treat the diseased condition, the underlying cause should be sought. If there are evidences of malaria, large doses of quinine will be of service. If it is thought to be pollen from certain plants, a change of climate will be the surest mode of relief. If the cause is one without assignable cause, it is always well to try such remedies as iron, quinine, arsenic, and iodide of pótassium, and other tonic and alterant remedies. Perhaps it is safe to say that no remedies give more uniform satisfaction than iodide of potassium and muriate of ammonia. These agents tend to promote the bronchial secretions, and patients find that as soon as the secretions are increased and expectoration established they are relieved. Hence I shall direct for this woman the following combination:

| | | |
|---|---|---|
| ℞ | Ammonii chloridi | 10 grains. |
| | Potassii iodidi | 8 grains. |
| | Syrupi zingiberis | 1 f. drachm. |
| | Aqu | q. s. |

M. Sig. For one dose, to be taken four times daily.

After two weeks the patient returned and reported that she has been entirely free from paroxysms since taking the above.—*Extractd from Clinical Lecture in Phil. Medical Bulletin.*

**Atropia for Vomiting in Pregnancy.**—J. W. Wade, M. D. (*in Medical Brief*) says: In vomiting in pregnancy I have been baffled in every remedy I could adopt except one, the sulph. atropia, hypodermically, one-sixteenth of a grain to one-half drachm of pure water in the arm, and belladon. oint. U. S. P. to the os uteri. For rigidity of os uteri this application of belladon. oint. with glycerine on a ball of cotton wool pressed against the os and kept there during six hours of the twenty-four, syringe well with warm water and castile soap and apply cosmoline or vaseline freely between applications of balladon. oint. I have usually succeeded. I have failed with oxalate cerium, bismuth and even champagne wine and ice, all of which are recommended and sometimes seem to relieve in vomiting in pregnancy, but atropia beats all.

**Corpulency Reduced by Diet.**—Dr. O. B. Campbell, Ovid, Mich., (*Physician and Surgeon*, January, 1881,) was consulted by a man weighing 304¼ pounds, concerning pain in the limbs and embarrassed respiration. The limbs were swollen, and pitted deeply upon pressure. There was a slight varicosity, and the urine contained traces of sugar. His normal weight was 180 pounds. Had taken various anti-fat preparations, and gained flesh all the time. By a diet of gluten bread, beef, eggs, tea and coffee without sugar, a minimum quantity of food and a saline cathartic at night, the weight was reduced to 290 pounds in seven days, and to 260 pounds in the first three months. He believes this case to exceed any previously reported in rapidity of reduction. The diet was recommended by the *Physician and Surgeon*, July, 1879.—*Medical Tribune.*

**Atropia for Hysteria.**—Dr. Leman, in *Medical Bulletin*, has found the hypodermic use of atropia effectual in the convulsions of hysteria:

The exact nature of hysteria has not been clearly made out. The tendency seems to be to give it a location in the nerve centres. Dr. H. C. Wood says "that many of the phenomena of hysteria are phenomena of inhibition, or lack of it." Rosenthal says "we must attribute a large part of the symptoms of hysteria to a congenital or acquired want of resistance on the part of the vaso-motor nervous system; that the disturbance of the brain must be attributed to reflex spasm of the cerebral arteries, and to the consequent cerebral anæsthesia." Dose one-twentieth to one-fortieth grain.

**Azotite of Ethyl.**—M. E. Peyrusson has published a remarkable paper on the use of this medicine as a prophylactic in pestilential and contagious diseases, and to purify contaminated localities. Azotite of ethyl in vapor possesses all the physical and chemical properties to attack all morbific products in the atmosphere. Its action is analogous to ozone, but more active in its effect by reason of the great variety of products it has to meet. It is no more unpleasant than ozone in odor, nor irritating in its action upon the tissues, and whilst it is impossible to use the latter practically, the azotite of ethyl can be used night and morning, placing some few grammes in a flagon in an impure room.—*Therapeutic Gazette.*

**Action of Bromide Potassium.**—Maragliano has found, by employing the method of cranial thermometry, that bromide of potassium, in doses of thirty to fifty grains, contrary to the usual theories of its action, causes a rise of temperature, at least on the outside of the cranium. This rise amounts on the average to about one degree centigrade; it reaches its acme in about an hour and a half and declines again in two or three hours. Simultaneously with this there appears a slight rise of two or three-tenths of a degree in the axilla. It is of course open to question, especially after the publication of Franck's researches, whether the temperature or the changes of temperature on the external surface of the head, represent approximately similar conditions of the brain or not. There does, however, seem to be some clinical evidence that there is a connection between external cranial temperature and intra-cranial changes, and if this rise following the ingestion of bromides really means an increase of cerebral circulation, then a popular theory of the action will have to be given up.—*Chicago Medical Review*, January 5th.

**Quinia from Coal Tar.**—Professor J. M. Maisch (*Amer. Journal of Pharmacy*, April, 1881, p. 176) states that the New York *Commercial Bulletin* notices a report current in that city that a Liberty street firm had applied for a patent for a process to manufacture quinia from coal tar. The firm has been interested with a chemist to accomplish this result for several years. Our readers have been kept informed of the results obtained by Skraup, Koenigs, Hess and others, in their endeavor to determine the exact composition of the cinchona alkaloids, which, when once known, will doubtless lead to their synthetical production. Of its importance there can be no doubt, when it is remembered that in 1879 $2,000,000 worth of cinchona bark was imported, most of which has, doubtless, been used in the manufacture of this indispensable alkaloid.—*Col. and Clin. Record.*

**Treatment for Rheumatism.**—DR. Cutter (*in Medical Brief*) says: With your permission I would like to call the attention of my professional brethren to the use of the fl. ext. of manaca in the treatment of rheumatism, both acute and chronic. I have used this drug in the treatment of a number of cases, and have never failed to give prompt relief. I prescribe it in ten drop doses three times a day before meals, for three or four days, then increase the dose to twenty drops. This I continue for three days. After all symptoms leave under this treatment, the pain and swelling subside rapidly. I never find it necessary to administer any remedy for the reduction of the fever.

**Membranous Croup.**—Dr. S. S. Green, of *Buffalo Medeical Journal*, on the treatment of membranous croup by the local use of perchloride of iron, reports the histories of seven cases, in which a concentrated solution of this salt had been thrown, in an atomized form, into the larynx, and, of the number, there were two deaths with five recoveries.

